# Comparison of Single-Treatment Efficacy of Bevacizumab and Ranibizumab for Retinopathy of Prematurity

**DOI:** 10.3390/children11080927

**Published:** 2024-07-31

**Authors:** Fumio Takano, Kaori Ueda, Yuko Yamada-Nakanishi, Makoto Nakamura

**Affiliations:** Division of Ophthalmology, Department of Surgery, Kobe University Graduate School of Medicine, 7-5-2, Kusunoki-cho, Chuo-ku, Kobe 650-0017, Japan

**Keywords:** retinopathy of prematurity, bevacizumab, ranibizumab

## Abstract

Background: Retinopathy of prematurity (ROP) is a significant cause of blindness in infants. Appropriate therapeutic intervention is essential because retinal detachment due to the progression of ROP is critical to visual function. The intravitreal injection of anti-vascular endothelial growth factor (VEGF) agents has been increasingly applied to inhibit the development and progression of ROP. In this study, we compared the efficacy of single intravitreal bevacizumab (IVB) and ranibizumab (IVR) injections for the treatment of ROP. Methods: A total of 39 eyes in 21 patients with severe ROP and IVB (15 eyes of 8 patients) and IVR (24 eyes of 13 patients) were retrospectively reviewed. Patient background, the severity of ROP, and the percentage of cases in which ROP regressed without additional treatment were compared between the two groups. Results: Patient background and ROP severity were not significantly different between the two groups. Recurrence was observed in one eye of one patient in the IVB group, and thirteen eyes in seven patients in the IVR group required additional laser photocoagulation, which was significantly different in the two groups (*p* < 0.01, Fisher’s exact test). In the IVR group, two eyes of two patients underwent vitreous surgery. Conclusion: Compared with IVR, IVB is likely to control the severity of ROP with a single treatment.

## 1. Introduction

Retinopathy of prematurity (ROP) is a vascular proliferative retinal disease that afflicts premature infants and threatens visual function. It is a major cause of infant blindness [1]. Although no comprehensive epidemiological study has been conducted in Japan, an epidemiological study in South Korea, which is in the same Asian continent and has similar demographics, revealed that the incidence of ROP decreased from 39.5% to 23.5% over 12 years from 2007 to 2018, whereas the incidence of ROP, which required treatment, also decreased from 4.7% to 1.8% [2]. Conversely, a recent epidemiological study in the United States, a leading developed country with accumulated epidemiological data on ROP, revealed that the incidence of ROP increased from 14.7% to 19.9% between 2000 and 2012, which was associated with an increase in the life-saving rate of extremely low-birth-weight and preterm infants [3]. The survival rate of premature infants increases with the availability of medical care as well as the rate of newborns experiencing complications from serious systemic diseases. Based on these reports, ROP occurs at a constant rate, despite the increased level of medical care. The results of epidemiologic studies differ worldwide because the incidence of ROP is affected by multiple factors, such as economic conditions or the level of perinatal medical management across countries [4].

During the process of ROP formation, neovascular vessels develop towards the avascular area of the retina, and proliferative membranes form as the disease progresses, causing tractional retinal detachment. ROP starts to regress at an average of 38.6 weeks of PMA and 44 weeks of remission in 90% of the cases [5]. However, if ROP deteriorates before spontaneous resolution is observed, appropriate therapeutic intervention is required. Retinal detachment especially can be critical to visual function [6]. Treatment strategies for ROP include surgical treatment, retinal photocoagulation, cryotherapy, and the intravitreal injection of anti-vascular endothelial growth factor (VEGF) agents. As discussed above, in situations where surgical treatment is required, retinal detachment has already occurred. Laser photocoagulation or cryotherapy of the avascular area are classic treatment strategies for severe ROP. These treatments reduce the oxygen demand and slow the progression of neovascular vessels of the retina [7]. The Early Treatment for ROP (ETROP) group established diagnostic criteria for the treatment of severe ROP with retinal photocoagulation [8]. Retinal photocoagulation increases severe myopia or astigmatism as the child grows [9].

In recent years, vitreous injections of anti-VEGF drugs have gained popularity as a new treatment strategy for ROP [6]. These agents suppress the growth of neovascular vessels and prevent ROP progression. The BEAT-ROP study revealed that the intravitreal injection of bevacizumab (IVB) was effective in the photocoagulation group in zone 1 plus ROP [10]. The RAINBOW study demonstrated that the effect of the intravitreal injection of ranibizumab (IVR) was similar to laser photocoagulation in both zone I and zone II ROP [11]. Based on the results of this study, IVR has received approval in more than 60 countries worldwide. Recently, aflibercept has been used as a new anti-VEGF agent; however, it could not show noninferiority compared to laser photocoagulation [12].

In addition, anti-VEGF agents themselves do not affect the retinal structure. Since no treatments exist for ROP that promote the development of visual function, the treatment that does not affect the intact retina is preferred. Fortunately, anti-VEGF therapy does not directly affect the structure or function of the retina, which is very important as a treatment option for ROP. Furthermore, most infants with severe ROP have other severe systemic diseases, so it is ideal to control the progression of ROP with a single treatment.

In the present study, we retrospectively compared the difference in recurrence rate and the need for additional laser photocoagulation therapy between IVB and IVR for patients with severe ROP.

## 2. Materials and Methods

This was a retrospective, observational study and the Institutional Review Board of the university hospital approved the study, which complied with the Declaration of Helsinki. Patients could withdraw consent at any time after reviewing information about this study on the hospital’s homepage as an opt-out choice, instead of receiving personal informed consent.

A total of 39 eyes in 21 patients, who were initially treated with either IVB or IVR for ROP from July 2018 to March 2021 at the university hospital, were included. IVB had been injected until November 2019, when ranibizumab received pharmaceutical approval in Japan. Since its approval, IVR has been used. Overall, 15 eyes in 8 eligible patients received IVB (0.25 mg/0.01 mL), and 24 eyes in 13 patients received IVR (0.1 mg/0.01 mL) per eye by vitreous injection.

Patients who received intravitreal injections in both eyes were included in the analysis. The following basic clinical information was collected for all patients: sex; birth weight; gestational age (GA); Apgar score at 1 min and 5 min; duration of ventilator-assisted respiratory management from birth; and the presence or absence of blood transfusion, sepsis, respiratory distress syndrome (RDS), and severe patent ductus arteriosus (PDA). Patient data at the time of intravitreal injection were collected, including the corrected weeks and body weight, zone and stage of ROP, presence or absence of aggressive ROP (A-ROP), and follow-up duration. The zone, stage, and type of ROP were determined based on the International Classification of ROP [13]. The diagnosis of severe ROP, which requires treatment intervention, was defined based on the ETROP study. Patients with zone 1 and any stage of ROP with/without plus disease, zone 2, stage 2/3 with plus disease and A-ROP were enrolled.

Intravitreal injection was performed under sedation. Sterilisation and cleanliness procedures were performed, such as those used in routine ophthalmic surgery. The injected eyes were dilated with 0.5% tropicamide/0.5% phenylephrine drops (Mydrin^®^-P ophthalmic solution, Santen, Pharmaceutical Co., Ltd., Osaka, Japan).

A 30 G needle was used for intravitreal injection, which was 1 mm from the corneal limbus and 4–6 mm vertically into the eye towards the fundus.

The primary outcome measure was the treatment success rate of IVB and IVR. The treatment success was defined as ROP remission without additional treatment following a single anti-VEGF intravitreal injection. To determine the effectiveness of the treatment and the necessity for additional treatment, patients underwent fundus examinations at least twice a week for the first month after injection. Thereafter, the frequency of examination was adjusted according to the severity of ROP.

Continuous variables were expressed as means ± standard deviations. Statistical analysis was performed using Fisher’s test and the Mann–Whitney U test, using freely available EZR software (http://www.jichi.ac.jp/saitama-sct/SaitamaHP.files/download.html; accessed on 19 April 2024). *p* < 0.05 was considered statistically significant.

## 3. Results

Table 1 shows the baseline characteristics for both groups.

In total, 15 eyes of 8 patients (4 males) were injected with bevacizumab and 24 eyes of 13 patients (5 males) were injected with ranibizumab. The mean GAs were 25.4 (±1.7) weeks in the IVB group and 25.1 (±1.8) weeks in the IVR group, with no significant difference between the groups (*p* = 0.942). The mean birth weight was 643.4 (±211.0) g in the IVB group and 622.3 (±208.0) g in the IVR group, which was not significantly different (*p* = 0.804). The mean postmenstrual age (PMA) at treatment was 35.5 (±3.4) weeks in the IVB group and 34.5 (±2.1) weeks in the IVR group with no significant differences (*p* = 0.664). The mean weight at treatment was 1493.4 (±395.8) g in the IVB group and 1284.2 (±367.9) g in the IVR group, with no significant differences (*p* = 0.238).

With respect to systemic complications, all cases had RDS, and the ratio of blood transfusion, PDA, and sepsis was not significantly different between each group.

Table 2 describes the data of ROP. 

The severity of ROP at the time of treatment was 5 eyes in zone 1, 10 eyes in zone 2, 6 eyes in stage 2, and 9 eyes in stage 3 in the IVB group. In the IVR group, the severity was 8 eyes in zone 1, 16 eyes in zone 2, 16 eyes in stage 2, and 8 eyes in stage 3. A-ROP was observed in one eye in the IVB group and two eyes in the IVR group. There were no significant differences in the severity of ROP in these zones, stages, A-ROP cases, or follow-up durations from the initial treatment between the two groups. In the IVB treatment group, only 1 eye in one patient exhibited deteriorated ROP, which required additional retinal photocoagulation, whereas 13 eyes required additional laser photocoagulation or surgical procedure in the IVR group. There were significantly more successful cases in the IVB group compared with the IVR group (*p* = 0.004, Fisher’s exact test). The mean durations from the initial anti-VEGF intravitreal injection were 12 days in the IVB group (only one case) and 50.6 (±37.1) days in the IVR group.

## 4. Discussion

In this study, there was a clear difference in the treatment effect of a single IVB and IVR on the recurrence rate of severe ROP. We speculate that this result may be due to the difference in half-times between IVB and IVR. The half-times of bevacizumab and ranibizumab injected into the vitreous of rabbit eyes differ by 4.3–6.6 days and 2.9 days [14]. In addition, in almost all cases, severe ROP requiring treatment develops after 30 weeks of PMA. Moreover, the average recurrence periods for bevacizumab and ranibizumab are 16 and 8.6 weeks, respectively [15]. From these results, when anti-VEGF agents are injected at 30 weeks of PMA, ROP recurrence occurs at 46 weeks with bevacizumab and 38 weeks with ranibizumab, respectively. Therefore, ranibizumab is predicted to have a higher risk of recurrence as monotherapy and a greater requirement for additional therapy. Not only our study but also several previous reports have shown that ranibizumab has a higher recurrence rate compared with that of bevacizumab [16,17,18,19,20].

Regarding the dose of bevacizumab, we treated patients with the lowest doses in previous studies because of concerns for unexpected systemic complications. As a result, we observed no recurrence of ROP in most cases, and a one-time injection was effective for controlling ROP. For the same reasons as bevacizumab, we treated patients with 0.1 mg/eye of ranibizumab. In the RAINBOW study, 0.1 mg/eye and 0.2 mg/eye of ranibizumab were compared and revealed that the dose difference was not associated with the efficacy of ROP treatment.

The largest limitation of this study is the small number of patients. In addition, this study did not evaluate late-stage complications, including the recurrences long-term of ROP. 

Another limitation is that we compared only the efficacy of the two anti-VEGF agents Recently, as the third agent for ROP treatment, a large clinical trial was performed to evaluate the efficacy of aflibercept [12]. Our study compared the effect of anti-VEGF drugs in a single treatment. Since aflibercept is expected to become a popular treatment for ROP, we believe it is necessary to compare and verify the efficacy of monotherapy with the three anti-VEGF agents—bevacizumab, ranibizumab, and aflibercept—in the future. 

## 5. Conclusions

For the treatment of ROP, IVB is more likely to control the course of ROP with a single treatment compared with IVR. Large prospective trials comparing the efficacy of multiple anti-VEGF agents are needed in the future.

## Figures and Tables

**Table 1:** Clinical Characteristics of the patients.

	IVB	IVR	*p* Value
male (%)	4 (50)	5 (62.5)	0.673 ^a^
GA at birth (wks, mean ± SD)	25.4 ± 1.7	25.1 ± 1.8	0.942 ^b^
weight at birth (g, mean ± SD)	643.4 ± 211.0	622.3 ± 208.0	0.804 ^b^
GA at treatment (wks, mean ± SD)	35.5 ± 3.4	34.5 ± 2.1	0.664 ^b^
weight at treatment (g, mean ± SD)	1493.4 ± 395.9	1284.2 ± 368.0	0.238 ^b^
Apgar Score at 1 min (mean ± SD)	3.3 ± 2.1	2.8 ± 2.2	0.505 ^b^
Apgar Score at 5 min (mean ± SD)	6.0 ± 2.5	6.5 ± 1.9	0.581 ^b^
duration of respirator use (days, mean ± SD)	117.4 ± 84.2	68.8 ± 26.4	0.277 ^b^
Blood transfusion (%)	8 (100)	11 (84.6)	0.505 ^a^
RDS	8 (100)	15 (100)	NA
severe PDA	0 (0)	1 (6.7)	1.000 ^a^
sepsis (%)	1 (12.5)	3 (23.1)	1.000 ^a^

GA: gestational age; wks: weeks; RDS: respiratory distress syndrome; PDA: patent ductus arteriosus; SD: standard deviation; ^a^ Fisher’s exact test; ^b^ Mann–Whitney U test.

**Table 2:** Severity of ROP of the patients.

	IVB	IVR	*p* Value
Zone1/2	5/10	8/16	1.000 ^a^
Stage2/3	6/9	16/8	0.184 ^a^
Plus sign (%)	15 (100)	24 (100)	NA
A-ROP (%)	1 (6.7)	2 (8.3)	1.000 ^b^
Success (%)	14 (93.3)	11 (45.8)	0.00492 ^b^
Duration from treatment (days, mean ± SD)	12	50.6 (±37.1)	NA
Duration of follow-up from treatment (days, median (IQR))	221 (123, 1546)	1210 (1114, 1421)	0.185 ^b^

IVB: intravitreal injection of bevacizumab; IVR: intravitreal injection of ranibizumab; ^a^ Fisher’s exact test; ^b^ Mann–Whitney U test.

## Data Availability

The data that support the findings of this study are available upon request from the corresponding author because they contain information that could compromise the privacy of research participants.
